# Mississippi Dental Association, 1895

**Published:** 1895-08

**Authors:** J. M. Walker


### Mississippi Dental Association, 1895.

Reported for the Dental Register by Mrs. J. M. Walker.

The Mississippi Dental Association met in annual session April 3, 1895, in Jackson.

The meetings were held in the Senate Hall of the State Capitol.

The President, Dr. W. E. Walker, of Pass Christian, occucupied the chair.

The meeting was opened with prayer by the Rev. Dr. Williams, of St. Andrew's Episcopal Church.

A brilliant address of welcome was delivered by the Hon. R. W. Banks, an address remarkable for bright wit and eloquent rhetoric, well sustaining the reputation of this very popular speaker for always saying the right thing at the right time and in the most charming and effective manner. After a brief but characteristic response by Dr. W. W. Westmoreland, of Columbus, the president read his

### ANNUAL ADDRESS.

Dr. Walker confined himself—after the usual words of greeting and congratulation—to suggestions of a practical character, for the consideration of the association.

Having reorganized, at the last meeting, under a charter and revised constitution, he urged upon the members the importance of familiarizing themselves with the provisions of the new constitution, and especially the conditions imposed by the charter. He said: "Allow me to suggest that every one who has not done so, should secure a copy of the charter, and study it that you may know and understand our powers, privileges and liabilities as a chartered body."

In view of the numerous applications annually made for beneficiary scholarships in the dental colleges by worthy young men of limited means, Dr. Walker reviewed the inducements offered by the colleges in Baltimore and Atlanta, the Northwestern College of Chicago and others; the scholarship prizes offered by the

Louisville, Ky., and the Ohio College, Cincinnati, the honor appointments of the New York College of Dentistry, etc.

He urged that special inducements be offered the young men who pass the board, to join the association at the same meeting. He said: "It is our duty to offer these young dentists every inducement to enroll themselves in our ranks that they may enjoy the advantages of association work, and thus prepare themselves to take the places of those who, year by year, are seen no more among us. By thus early placing in their hands the Constitution and Code of Ethics, to which they must subscribe on joining the association, many of them may be led into and kept in the right path, who, if allowed to go back to their homes with only the promise—and no doubt the honest intention—of joining us when better able to meet the expense, might, in the busy strife of competition, without this restraining influence, go so far astray that we might never win them back to the path of professional honor and integrity."

He called the attention of the association to the importance of efforts to secure, through the Legislative Committee, such amendments to the dental law as shall secure to dentists exemption from jury duty and militia duty. Also that an effort be made to secure the enactment of a law making malpractice (in dentistry and if possible in medicine and pharmacy as well), a misdemeanor, the penalty of the same to be forfeiture of license to practice. The restriction of the promiscuous extraction of teeth, and the injection of nostrums for so-called painless dentistry is also in the hands of the Legislative Committee.

A communication from the Louisiana State Dental Society looking to a Tri-State meeting of the Louisiana, Texas and Mississippi Dental Associations in the spring of 1896, was read and given appreciative consideration. The charter of the Mississippi Association requiring that all meetings be held "within the State," makes the proposed meeting impracticable and the invitation was declined.

Drs. C. L. Alexander, Charlotte, N. C.; D. R. Stubblefield and J. Y. Crawford, Nashville, Tenn.; T. P. Hinman and Frank Holland were guests of the association for the first time and were elected to honorary membership. Dr. Wm. Crenshaw, an old friend and honorary member of the association was also present.

The first paper read was by Dr. J. P. Broadstreet, Grenada, on the subject of "The Care and Developtment of the Teeth."

The paper was a powerful arraignment of the negligence and indifference of the dental profession toward little children, and the necessity for the education of parents and the public, teaching the importance of caring for the teeth of their children.

The paper received very earnest discussion. Drs. Stubblefield and Crawford were especially eloquent upon this topic. Dr. Westmoreland urged the importance of guarding children against eruptive fevers while the teeth are in the formative stage. Dr. Crawford spoke of the injurious effects of the rubber nipple of the nursing bottle upon the tissues of the gums, many babies having it in their mouths almost continually. He considers the inventor responsible for many of the deaths that occur among teething children.

He attributes a large proportion of infantile mortality during that period so dreaded by mothers and nurses while "cutting" the "stomach" and "eye-teeth" as due to keeping the child too long at the breast or the bottle instead of weaning it after the eruption of the first baby molars—nature's indication of the need of solid food—the benign influence of function giving the exercise needed for the development of the jaw to make space for the broad crowns of the cuspids.

Without this exercise—denied to the child kept at the breast or on the bottle—the jaw remains undeveloped, and the cuspids are bound down between the baby laterals and first molars; they cannot erupt, the child has convulsions and dies.

Ages of experience have taught mothers and nurses that this is a period in dentition to be looked forward to with apprehension of serious trouble, but why this should be so they have never been taught.

Calling the Vice-President, Dr. Frank Smith, Grenada, to the chair, Dr. W. E. Walker read the next paper entitled: "A Unique Case in Amputation of Tooth Roots."

He considered the case unique from the fact that with no caries anywhere in the mouth, the pulps of the first four molars had died and the teeth abscessed, one root of each of these molars being entirely devoid of vital attachment, the pericementum being destroyed over the whole surface of the necrosed root, with pus discharging around the gingival margin. There had been more or less discharge from the abscesses for twenty years when the patient first presented, in the spring of 1892. At that time the chronic inflammation had developed an ugly-looking tumor on the gum of the posterior root of the lower first molar. Examination revealing the condition of the root, as described, amputation of the root was decided upon. The perfect crown was opened into from the sulsus, the putrescent contents of the pulp chamber and the roots removed and when ready the anterior canals were filled. The posterior root was then amputated on a line above the gum margin to the point of bifurcation, the pulp chamber filled with gutta-percha which was pressed out through the opening at the point of amputation and smoothed off below with a warm instrument. The morsal opening was filled as usual, and the patient left for his Northern home without treatment of the tumor or the three other abscessed first molars.

Three years later, in March of the present year, the patient again visited the Gulf Coast and reported requesting similar treatment of the other three first molars, the operation described having proved a perfect success. The tumor had disappeared, the abscess had healed, the socket had not only filled up but the tissues had built up under and around the projecting posterior portion of the crown, so that only probing with an instrument revealed the absence of the entire posterior root.

The operation was repeated, the posterior root right lower, and the lingual root of each of the superior first molars being similarly necrosed. The roots were amputated and the opening filled as described, with every prospect of saving four good molars instead of giving the patient a plate, which would have been necessary had the teeth been extracted.

The paper was discussed by Drs. Holland, Stubblefield, Crawford, Alexander and others. In the discussion the importance of saving as much of the tooth substance as possible, especially on single-rooted teeth, was urged by Drs. Stubblefield and Crawford. Dr. Crawford considers a treatise on dental sugery incomplete which does not include this operation.

Dr. Walker described the operation, as performed by him, as follows:

When it is decided at what point the root shall be excised, which in molars is generally about $\frac{3}{32}$ of an inch from the cemento-enamel line, a hole is drilled, slanting apically to the point of bifurcation; this is followed by others on either side. With a fissure-drill the holes are then connected on a plane and cut through to the circumference of the root, which is then easily removed with an elevator or delicate pair of forceps, without disturbing the tooth.

Dr. Holland thought the operation more easily described than performed.

The second and part of the third day of the meeting was devoted to clinics.

Dr. C. L. Alexander, Charlotte, N. C., the special clinician for this meeting, constructed a piece of bridge-work by an original method, which he terms a

SUSPENSION DENTURE,

A method by which the teeth are not subjected to cutting or banding, the denture being supported by short screw points in the lingual portion of the natural teeth. In the case operated on at the clinic, the superior left lateral and right central incisors were to be supplied. A narrow band, or skeleton attachment, was adjusted to the lingual surface of the right lateral and left central with arms extending to each of the cuspids. On the band a hollow receptacle was constructed partially filling each of the vacant spaces, with holes in the anterior walls to receive the long pins of bridge-work facings which were secured in place by bending the pins inside and filling the hollow with cement with a facing of amalgam to protect the cement from the fluids of the mouth.

The piece was constructed upon a metal model having pins corresponding to the screw points the piece when finished being simply slipped into place, the pins in the lingual surface of the teeth passing through correponding holes in the skeleton attachment.

Dr. T. P. Hinman demonstrated his special methods of securing perfect adjustment of the Logan crown and a new method of banding the Logan crown with the aid of a little screw-press of the Hollingsworth contouring method, using 22 k. gold of 28 or 29-guage for the band, reducing it to the necessary thinness under the gum margin.

Dr. Crenshaw demonstrated practically the method of bridge repair in the mouth, using the Bryant method and outfit.

Dr. Crawford demonstrated an original method of restoring broken incisors, with porcelain tips, grinding the tip from a porcelain tooth—preferably of English body because of susceptibility to high polish after grinding. In the present clinic a central incisor broken across the crown and having large gold approximal fillings was cut squarely across both gold and tooth substance, and a lower incisor adapted as porcelain tip, being placed transversely, and so reversed as to place the lengthwise pins of the porcelain tooth in holes drilled perpendicularly in the tooth, on either side of the living pulps—and preferably in the gold filling where the case admits. Other clinics were made by members of the association.

At the night session of the second day Dr. D. B. McHenry, of Grenada, read a paper on Metallurgy. The arguments of the paper being in favor of a return to the older methods of metal work, prosthetic dentistry in vogue before this branch was debased by the introduction of rubber plates.

Dr. J. B. Askew, Vicksburg, read a paper on "Dental Education" which led to a lengthy discussion covering education, legislation and ethics.

Dr. J. Y. Crawford spoke of the intimate connection between dental education and dental legislation and of the chaotic state of the latter owing to the effort to make the dental law cover every possible contingency. It all hinges on the simple question: What is right? What is wanted is equal and exact justice to all. He thought that every college diploma should include a solemn oath, before a notary, to abide by the Code of Ethics. Then, if a man violates his word, he perjures himself, and no perjurer can have the confidence of any community.

Drs. Holland and Crenshaw spoke of the contradiction involved in some of the States, in which a college chartered under the laws of the State confers a diploma which a Board of Examiners, created by the same law, refuses to recognize.

Dr. W. T. Martin, Yazoo City, spoke of the difficulties attending the efforts to secure the enactment of the earlier dental laws, which are the basis of all subsequent legislation. Those laws were the best that could be had at that time, but are far from being what is wanted and needed now, in view of the great advances that have been made all along the line.

Dr. W. E. Walker, calling the vice-president to the chair, spoke of the insufficiency of the degree conferred by the dental colleges, and of the license granted by the State Boads. He said: "I do not think we should be confined in name (as we certainly are not in practice) to surgical operations upon the teeth. * * We cannot practice our profession successfully if we limit ourselves to treating merely the teeth, without, in very many cases, treating the surrounding tissues also. It often becomes our duty to treat diseases of the antrum, to treat abscesses after their are no teeth left, to treat necrosis of the maxillaries.

He said: I do not believe that we have any legal right, under our license to practice dentistry, to treat many of the diseases which nevertheless we do, and must treat, to make successful the more purely dental services we render." He said that he had treated some cases in conjunction with the physician, and in some cases (notably of abscesses) he had had to take a bold stand in opposition to the physician, and he thought he would have found it difficult to prove that he was practicing dentistry and not medicine, though he has no license—no legal right—to practice medicine other than the dental specialty.

Dr. Walker then suggested for consideration the degree of S.D.—Stomatologiæ Doctor—to obtain which, the degree M.D. as well as that of D.D.S. should be a prerequisite, the degree representing the education which a man receives both in medicine and dentistry, putting the mouth-doctor on a par with the eye-doctor as a specialist. The attainment of this degree would require a course of probably four years, the courses in medicine and dentistry running *pari passu*, as is the case in the dental and medical departments of some of the universities, a few hours each day being devoted, during the entire course, to the development of skill and manipulative ability—the education of the fingers so essential to the dentist.

In the discussion of the report from the committee appointed to consider the president's address, DR. J. B. ASKEW related some incidents in his experience, relative to jury duty. On one occasion he was forced to serve on a jury on which he was the only white man; serving on a criminal jury with eleven negroes. On another occasion he was summoned at a time when he had three critical cases on hand. One, a young lady who had closed her school fifty miles away, to come to the city for the treatment of three abscesses; another, an abscess which had opened on the chin (the result of a railroad accident), with very offensive discharge and acute suffering requiring attention twice a day; the third, a gentleman coming on that day from a distance by railroad for pressing services. He sought the district attorney, who said there was nothing in the law to exempt him. Finally, through personal influence with the counsel on the other side, and the friendship of the judge he get off. Such cases as these are convincing evidence that dentists should be exempt from jury duty.

DR. J. P. BROADSTREET said that physicians are exempt from privilege-tax on the ground that they do so much charitable work, but he thought that argument applied with equal force to the dentist. Dr. Frank Smith added that the dentist not only does quite as much charity work as the physician, but is also at expense for material.

After an interesting discussion the following resolutions, offered by DR. J. B. ASKEW, were adopted:

"*Resolved*, That it shall be the duty of the Legistative Committee to urge upon the Legislature the importance of exempting dentists from jury duty, militia duty and the payment of privilege tax.

*Resolved further*, That members of the association are requested to see the members of Congress representing their district and urge upon them the necessity of appointing dentists in the army and navy."

Dr. Askew called attention to the fact that the Confederate Government was the only government in the world that appointed dentists to the army and navy, but he was satisfied it could be done again if properly urged before Congress.

As a new governor will be elected and go into office before the next meeting of the association, one of whose first duties will be the appointment of a new Board of Dental Examiners, the following dentists from different sections of the State were selected by the association for recommendation to the governor in his selection of the five members of the board: Drs. Geo. B. Clement, W. E. Walker, P. H. Wright and J. A. Warriner of the list recommended to Governor Stone and appointed by him on his first board; also Dr. L. A. Smith in place of Dr. Geo. B. Rembert, resigned from the association and left the State; also Drs. D. B. McHenry, L. G. Nesbit, J. B. Askew and E. E. Spinks constituting the present board, D. Birdsong, the fifth member, having resigned. The vote of the association was unanimous in favor of this list.

Dr. W. E. Walker read a paper giving the history of three cases of abscesses on the roots of teeth containing vital pulps.

Drs. W. T. Martin and L. A. Smith reported cases of the same nature.

Dr. J. P. Broadstreet said he had always considered alveolar abscess a certain indication of dead pulp and had never encountered a case such as those described in the paper. In reply to questions Dr. Walker stated that in the cases he described the gingival margin was intact, and in the second case the sinus treated after the removal of serunal calculus from the apical portion of the side of the root, through an opening made by distending the sinus with cotton for a day or two, the removal being facilitated by the application of a drop of trichloracitic acid.

Several papers were read by title only and ordered published in the proceedings for want of time for discussion.

The papers were, on "Inflamed and Abscessed Teeth, and Methods of Treatment," by Dr. L. A. Smith, Port Gibson.

"Fermentation of Carbo-hydrates as the Principal Source of Acidity in the Human Mouth," by Dr. P. H. Wright, Senatobia, and "A Few Remarks on Fillings," by Dr. T. C. West, Natches.

The election of officers was unanimous, but one candidate being put in nomination for each office, as follows:

Dr. T. C. West, Natchez, President; Dr. Frank Smith, Water Valley, Vice-President; Dr. L. H. Jeffries, Natchez, Secretary; Dr. C. C. Crowder, Kosciusko, Treasurer.

The association adjourned to meet in Jackson, in 1896. The date to be announced by the Executive Committee.

